# Displacement Self-Sensing Active Magnetic Bearing Drives—An Overview

**DOI:** 10.3390/s25206481

**Published:** 2025-10-20

**Authors:** Yiling Yang, Yunkai Huang, Fei Peng, Yu Yao

**Affiliations:** School of Electrical Engineering, Southeast University, Nanjing 210096, China; 220232886@seu.edu.cn (Y.Y.); pengfei@seu.edu.cn (F.P.); yuyao@seu.edu.cn (Y.Y.)

**Keywords:** active magnetic bearing (AMB), displacement self-sensing, demodulation, parameter identification

## Abstract

Displacement self-sensing active magnetic bearings (AMBs) have garnered significant attention from both academia and industry for their potential to reduce cost, enable system integration, and enhance reliability. While numerous self-sensing methodologies have been researched, the field lacks a unified framework for discussing their theoretical foundation and practical applicability. This paper analyzes and summarizes various displacement self-sensing methods, deriving the underlying principles and essence of these techniques, and clarifying the intrinsic interconnections of different schemes. The process of self-sensing is constructed through two steps: online inductance estimation and electromagnetic inductance modeling. A novel framework is then proposed, categorizing online inductance estimation, with dedicated discussion on modeling and handling critical nonlinearity like magnetic saturation and the eddy current effect. Furthermore, this review conducts a systematic comparative analysis, evaluating prevalent schemes against key performance metrics such as robustness, stability, signal-to-noise ratio (SNR), and system complexity. Finally, persistent challenges and future research trends are discussed. This review provides a valuable reference for both researchers and engineers when selecting and implementing self-sensing technologies for AMB systems.

## 1. Introduction

With increasingly stringent energy-efficiency targets, active magnetic bearings (AMBs) have emerged as a prevalent and promising technology, demonstrating outstanding performance and potential in multiple industrial rotating machines, such as compressors, turbines, and generators [1,2]. AMBs offer significant advantages over other contact and non-contact bearing technologies, including adjustable dynamics, high load capacity, and the omission of lubricants. Additionally, they can operate in extreme environments such as a vacuum and high-temperature conditions. Traditional AMB systems rely on displacement sensors to measure rotor displacement and achieve feedback control. However, AMB systems equipped with displacement sensors have the following shortcomings in practical applications:

1. Cost and reliability: Displacement sensors used in magnetic bearing systems include inductive displacement sensors and eddy current displacement sensors. In high-precision applications, eddy current sensors are often preferred due to their high resolution and fast response characteristics. However, eddy current sensors are costly, which increases manufacturing costs, and they can reduce the reliability of the system.
2. Failure of position detection in bending mode: When the rotor speed approaches the bending critical speed, the system may exhibit the flexible mode [3,4]. Due to the non-collocation arrangement between axial sensors and actuators, deriving the accurate displacements through a linear transformation is impossible. This non-collocation significantly increases the complexity and uncertainty under ultra-high rotating speed.
3. Integration limitations: Displacement sensors are typically arranged along the rotor’s axial or radial direction, thereby increasing axial length or occupying radial space, which increases volume and limits the high integration design of the system.

Displacement self-sensing technology provides a novel approach that does not require displacement sensors by directly estimating rotor displacements by processing signals within the actuators. Compared to traditional sensor-equipped systems, self-sensing offers significant advantages in terms of improving reliability, reducing manufacturing costs, minimizing size, and enhancing integration [5,6,7]. As a result, self-sensing technology has become an important research direction in AMBs and has attracted widespread attention.

The displacement self-sensing technology for active magnetic levitation bearings was first proposed by Vischer, a scholar from the Swiss Federal Institute of Technology in Zurich (ETH Zurich), in 1988 [8]. Vischer’s method was based on the Luenberger observer scheme for the linearized state-space model of the equilibrium point in a single-degree-of-freedom (1-DOF) magnetic levitation system (MagLev) [8,9]. Subsequently, Mizuno et al. investigated the stability of magnetic levitation systems without displacement sensors [10], and provided an analysis of the stability differences between self-sensing systems using full-dimensional observers and reduced-dimensional observers [10,11,12]. Morse et al. compared the upper bounds of the compensating sensitivity functions for two magnetic levitation control systems—one equipped with displacement sensors and the other based on a linear observer—using the small gain theorem and insensitivity function robustness theory. They concluded that the robustness and stability of the MagLev system based on a linear observer are significantly lower than those of the system equipped with displacement sensors [13].

As numerous experimental studies found that the robustness estimates above were overly conservative [14,15,16], Maslen and Montie from the University of Virginia revised the aforementioned view using a linear periodic model and analyzed the robustness limits of self-sensing AMB systems, with conclusions closely aligned with experimental results [14,17]. The LP model accounts for the switching ripple effects of the PWM power amplifier itself, indicating that utilizing the periodic ripple components in voltage and current can enhance the robustness of self-sensing technology. This is also one of the foundational theoretical studies for AMB self-sensing schemes based on demodulation methods. In recent years, various displacement estimation methods based on advanced control system theories such as nonlinear observers and adaptive filters have been proposed [18,19]. Since the single-degree-of-freedom MagLev system is inherently a third-order, nonlinear, highly uncertain system with multiple mismatched disturbances, the design of nonlinear observers must prioritize performance metrics such as convergence and robustness. Mystkowski et al. designed a sliding mode observer that leverages the insensitivity of sliding mode control to matched disturbances to address model uncertainty, and incorporated a magnetic circuit nonlinearity correction mechanism [18], but the experimental results were unsatisfactory. The Kalman filter displacement estimator optimized using a particle swarm algorithm proposed by scholars from Jiangsu University was successfully applied in a laboratory setting for a three-pole, five-degree-of-freedom AMB. However, the Kalman filter algorithm for the state transition model of a high-order (15th-order) AMB poses significant computational challenges for digital controllers [19].

Another technical approach for self-sensing AMBs is parameter estimation, which involves deriving displacement through signal detection or system identification algorithms using the physical model of electromagnet inductance–displacement. Parameter identification methods emerged in the early 1990s and can be categorized into demodulation methods based on signal analysis and parameter identification methods based on dynamic models [20,21]. These methods generally rely on high-frequency (HF) signals. If a linear power amplifier is used, an additional high-frequency power supply is required to generate the injection signal. Conversely, the maturity of modern power electronics technology has enabled the widespread use of PWM switch amplifiers. By utilizing the switching ripple inherent in PWM, rotor displacement information can be obtained without applying an injection signal.

Depending on the type of information being analyzed, demodulation methods can be categorized into amplitude demodulation [6,22,23,24], frequency demodulation [20,25,26,27], and phase demodulation [28]. Displacement self-sensing based on amplitude demodulation typically involves three signal processing stages: bandpass filtering, peak detection, and low-pass filtering. Early researchers used analog circuits to implement signal demodulation [22,29,30,31]. With the improvement in digital microcontroller processing capabilities, digital signal frequency analysis techniques, including Fourier transform [32], digital phase-locked amplifiers [24], current change rate detection, wavelet transform [33,34], Hilbert transform, etc., have gradually replaced the rectification and low-pass filtering stages of analog circuits, and together with analog anti-aliasing filters, form a mixed analog–digital demodulation scheme. Compared to pure analog schemes, mixed analog–digital schemes have higher hardware complexity, but they require higher processor computational capabilities, consume more computational resources, and have higher software complexity, though they offer more flexible configuration and implementation options. To address the dependence of PWM ripple amplitude on the duty cycle and the PWM sideband effects caused by the dynamic duty cycle [35,36], Zhu and Tang et al. proposed a compensation strategy using Butterworth wideband bandpass filters (BPFs) and eCAP real-time duty cycle detection [23].

In [34], a dual orthogonal spline wavelet algorithm to replace the traditional BPF is employed, addressing the issue of non-uniform convergence caused by the Gibbs effect in non-stationary characteristics of non-periodic waveforms. Reference [25] utilizes a hysteresis PWM power amplifier to achieve detection based on the relationship between inductance and PWM ripple frequency under fixed current ripple magnitude conditions; however, such methods exhibit poor dynamic performance during current reference transients and are difficult to resolve. The phase demodulation method based on orthogonal injection signals proposed in [28] has too low sensitivity and high parameter dependency, making it difficult to apply in practice. Additionally, some scholars have proposed a nonlinear parameter estimation method that uses an inductance model to estimate the current, then constructs a closed-loop inductance estimator using the current estimation error, and applies the Popov circle criterion to select parameters to ensure algorithm stability [21,29,37,38]. Parameter estimation methods based on system identification typically involve collecting PWM current ripple, constructing parameter estimation formats using discrete dynamic models driven by switching voltages, and identifying unknown inductance using the least squares method [39,40].

In addition to the development trend of digital alternatives for demodulation schemes, the refinement of inductance models is also a key research focus in demodulation-based AMB displacement self-sensing. Traditional inductance–displacement estimation models are based on the assumption of a linear magnetic circuit, ignoring the effects of magnetic field saturation, eddy current effects, and cross-coupling between radial displacements. To account for magnetic saturation in displacement self-sensing strategies, existing methods include magnetic circuit polynomial fitting [22,31,41], FEM-based precise numerical optimization of magnetic network analysis [42,43], weight-based magnetic saturation avoidance methods [44], and data-driven and machine learning approaches [45]. Meeker et al. studied the magnetic bearing pole model, considering eddy current effects, and proposed a complex permeability and equivalent cascaded RL network model [46]. Methods for compensating eddy current effects include the complex permeability lookup table method [42], RL network inversion method [47], dynamic compensator method [48], and current offset correction method [49], among others.

In addition, some studies have incorporated similar physical sensors into power amplifiers by improving their structure. In [36], a dedicated sensing winding to the magnetic bearing core is added to amplify ripple current, and an RLC passive filter is incorporated to improve PWM noise. In [50], the middle tap of the electromagnet coil is extracted as an independent sensing subsystem separate from the power section, and a resonant network is designed to enhance the sensitivity of displacement self-sensing. This paper focuses on the theory and algorithms of displacement self-sensing based on general amplifier topologies, so such structure-inherent displacement sensors are not discussed here.

As displacement self-sensing schemes become more diverse and research deepens, there are still some questions that need to be answered. First, regarding the relationship between various magnetic bearing displacement self-sensing methods—whether they are formally opposed or compatible, and whether their theoretical analyses converge—there is currently a lack of clear discussion on how to integrate them into a comprehensive research framework. Additionally, the performance differences between these methods in terms of their fundamental principles and practical applications have not yet been uniformly evaluated within a single system model. Therefore, this paper aims to address these issues through a technical review. The remainder of this paper is structured as follows: Section 2 outlines the comprehensive research framework for displacement self-sensing and summarizes the basic research approach; Section 3 discusses the first part of the research framework, namely how to establish a physical model of the electromagnetic coil inductance and air gap; Section 4 and Section 5, respectively, discuss inductance parameter estimation algorithms from the perspectives of demodulation methods and least squares parameter identification; and Section 6 provides a summary of existing methods and an outlook on future trends.

## 2. Principles and Research Frameworks

### 2.1. State Observer Approach

To achieve real-time estimation of the state of magnetic levitation bearings, the state observer in modern control theory is a potential approach. The state space equations for a 1-DOF magnetic levitation system can be obtained as

(1) $$\begin{array}{c} \dot{x}=v\\ \dot{v}=\frac{k_{s}}{m}x+\frac{k_{i}}{m}i_{c}+\frac{F_{d}}{m}\\ {\dot{i}}_{c}=-\frac{k_{i}}{L_{0}}v-\frac{2R}{L_{0}}i_{c}+\frac{1}{L_{0}}u+d \end{array}$$

where *x* is the displacement, *v* is the velocity, $i_{c}$ is the control current that is superimposed on the bias current, *u* represents voltage, *m* is the mass of the rotor, $L_{0}$ is the inductance under nominal gap, *R* is the resistance of the coil, and $F_{d}$ and *d* are disturbances. The states of the above model are fully observable, and thus the Luenberger observer can be used.

**Figure 1:**
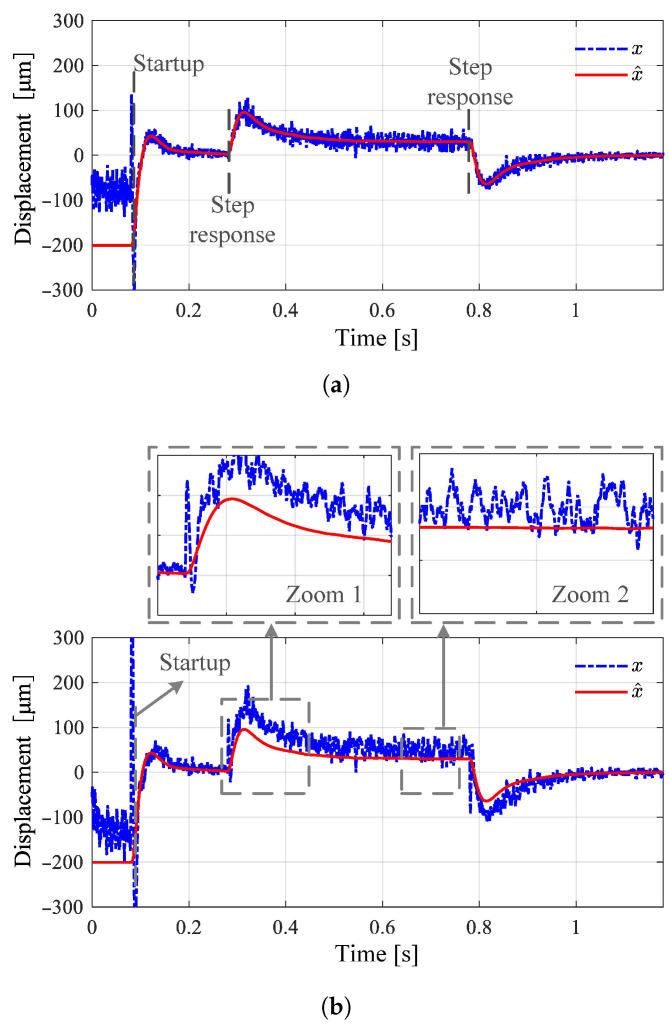
Simulation results of observer-based displacement estimation: *x* and $\hat{x}$ are measured and estimated displacement relatively. (**a**) Parameters match. (**b**) Parameters mismatch.

The observer method is only theoretically feasible and has numerous insurmountable weaknesses in practical applications. Figure 1 shows the simulation results of displacement estimation based on the Luenberger observer, where (a) represents the case of parameter matching and (b) represents the results of parameter mismatch. These results can be used to explain the reasons for the failure of the observer method. First, when there is no initial suspension, position detection fails because the system model does not satisfy the state equation shown in Equation (1). Therefore, the prerequisite for this method is stable suspension, which in turn requires displacement values as feedback, making self-starting impossible. After the suspension body floats, since the mathematical model is only accurate in the local region near the equilibrium point, the greater the deviation from the equilibrium point, the larger the error and noise. When model parameters have a 30% error, as shown in Figure 1b the estimated displacement values deviate significantly from the true values. In actual magnetic bearing systems, parameters exhibit strong uncertainty and multi-source disturbances, so the accuracy, disturbance resistance, and robustness of this method are insufficient to meet practical application requirements [16,17,51,52].

### 2.2. Parameter Estimation Approach

**Figure 2:**
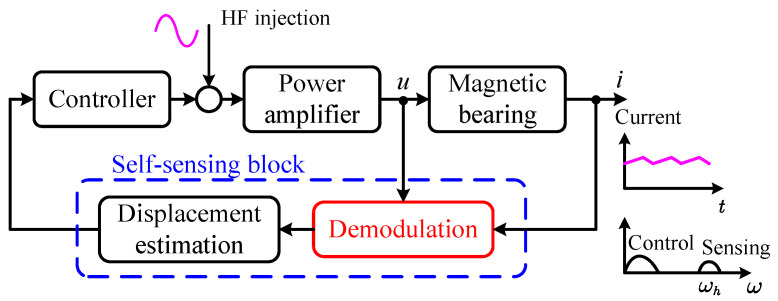
Block diagram of the parameter estimation method.

Parameter estimation is a two-stage method, which firstly uses the signal from the magnetic bearing coil to estimate the inductance, and then estimates the displacement according to the relationship between inductance and the air gap. The signal used to extract displacement is typically a high-frequency signal far from the control frequency. For convenience of expression and to reflect the nature of these high-frequency signals, they will be referred to as “sensing signals” in the following text, and their frequency will be referred to as the “sensing frequency.” The block diagram of the self-sensing system based on the parameter estimation method is shown in Figure 2.

For a 1-DOF MagLev, the inductance expression of the electromagnetic coil can be obtained using the virtual displacement method [1] as

(2) $$L=\frac{\mu_{0}A_{p}N^{2}}{2g+l_{m}/\mu_{r}}$$

where $\mu_{0}$ and $\mu_{r}$ are the permeability of air and the iron core, $A_{p}$ is the area of one magnetic pole, and *g* and $l_{m}$ are the effective length of the air gap and the core.

There are two methods for estimating inductance. One method starts from a high-frequency model, using the high-frequency voltage and current components to obtain impedance, and then deriving inductance. This method is called demodulation. The other approach is based on parameter identification theory, using the transient model of the actuator to construct a parameter identification equation in the form of overdetermined linear equations. The solution is then determined using the least squares method to minimize the squared error. This method is referred to as the identification method.

### 2.3. Framework of Displacement Self-Sensing

**Figure 3:**
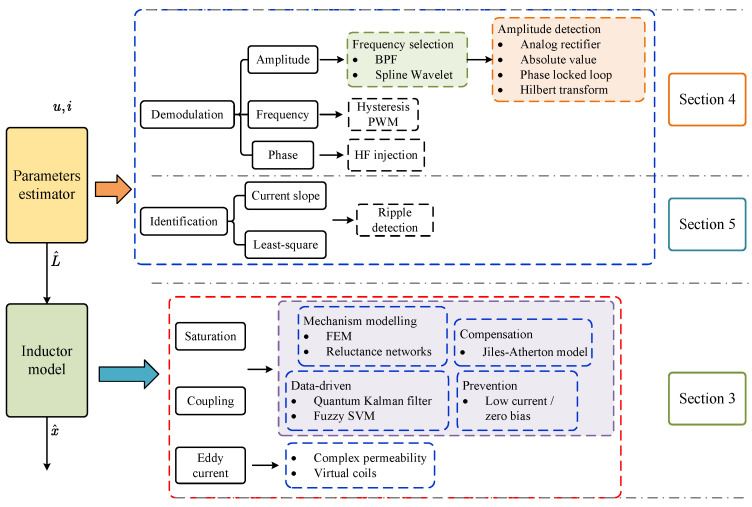
Overall framework diagram of technical routes of self-sensing.

The failure of the state observer method has been analyzed and verified. Magnetic bearing displacement self-sensing is mostly based on parameter estimator schemes. A general framework of the technical routes for displacement self-sensing is demonstrated in Figure 3 and summarized as follows:

1. Construction of the inductor physical model: This is used to establish the mapping relationship between the air gap and the inductor. The simplest form is shown in Equation (2), and the ferromagnetic nonlinear terms can be neglected. In practical applications, non-ideal factors should be considered based on actual conditions and performance requirements, including magnetic circuit saturation, eddy current effects, leakage inductance, and magnetic circuit coupling in multi-degree-of-freedom magnetic bearings.
2. Inductance estimator based on demodulation or least squares identification: The primary task of demodulation is to extract the sensing signal and its amplitude or envelope from the total current. There are numerous practical solutions, including analog circuits and digital signal processing algorithms, that can accomplish this step. To avoid rank deficiency in the identification equations, the least squares method requires the injection of additional dynamic signals. Therefore, it can be divided into single high-frequency signal injection and PWM ripple detection methods.

For the completed displacement self-sensing system, testing and evaluation are necessary to determine its performance. The evaluation should include the displacement estimation accuracy, signal-to-noise ratio, estimator dynamic response speed, and robustness and stability as defined in the ISO-14839 international standard [51] which is listed in Table 1.

## 3. Inductance Model of Magnetic Bearings

**Figure 4:**
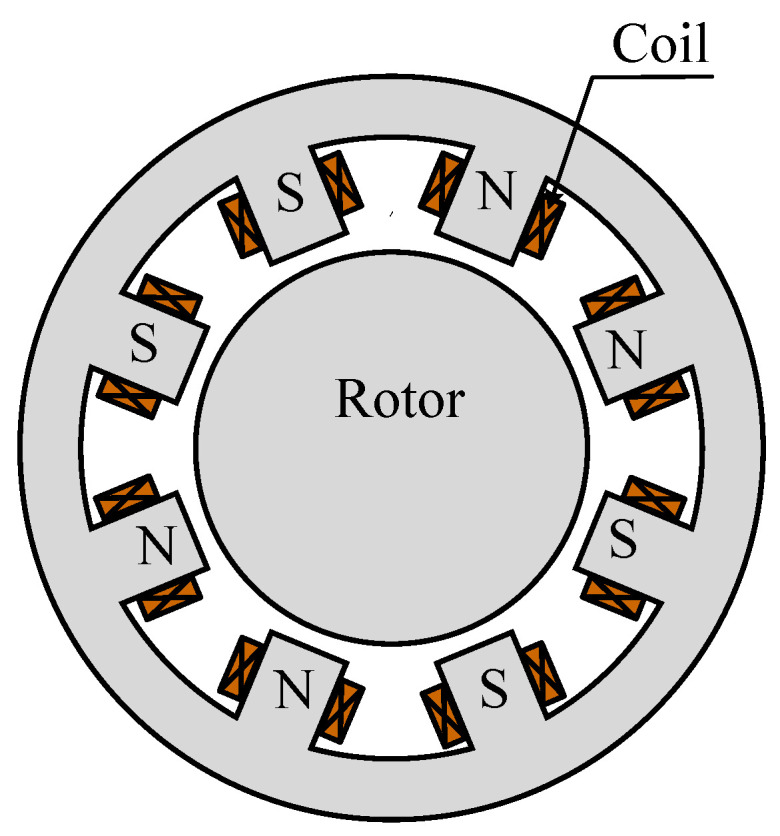
An 8-pole heteropolar magnetic bearing.

Magnetic bearings are subject to many non-ideal factors, including leakage inductance, magnetic saturation, eddy current effects, and magnetic circuit coupling in multi-degree-of-freedom magnetic bearings. By utilizing the radial eight-pole heteropolar magnetic bearing structure whose cross-sectional view is shown in Figure 4, the magnetic flux generated by the *x*-axis coil exhibits a marked inability to link with the *y*-axis coil, which weakens the magnetic circuit coupling between the *x* and *y* axes. In current-biased magnetic bearings, the bearing core always operates in the first quadrant, exhibiting a local hysteresis loop, which has a limited impact on displacement estimation. Therefore, this section is principally concerned with the description and solution of saturation and eddy current.

### 3.1. Modeling and Online Correction of Saturation

**Figure 5:**
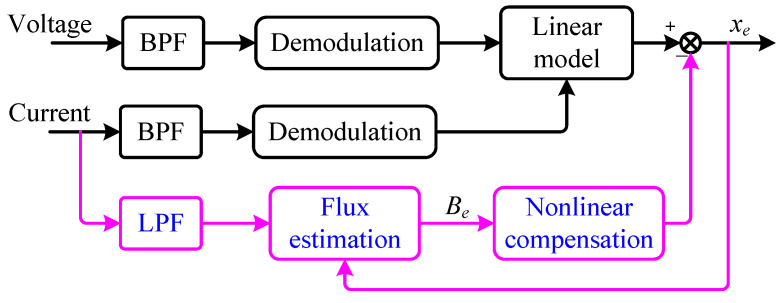
Block diagram of self-sensing with nonlinear compensation.

The Preisach model [53,54,55,56] and the Jiles–Atherton model [57] are two prevalent mathematical models for modeling saturation. When the magnetic flux density *B* exceeds a specific threshold, the relative magnetic permeability $\mu_{r}$ of ferromagnetic materials decreases sharply, causing the ferromagnetic resistance to become non-negligible compared to the air gap resistance. A simplified approach for describing magnetic saturation involves employing a polynomial function to represent the nonlinear behavior of the magnetic circuit. In [41], the relationship between the relative permeability $\mu_{r}$ and magnetic flux density *B* is approximated by a polynomial function as

(3) $$\frac{1}{\mu_{r}}=a_{m2}B^{2}+a_{m1}B+a_{m0}$$

where the coefficients $a_{m2}$, $a_{m1}$ and $a_{m0}$ are obtained through offline fitting using measured data, enabling online compensation for nonlinear errors. The block diagram of the displacement estimation section combined with nonlinear compensation is shown in Figure 5.

As can be seen from the block diagram, there is a feedback loop in the compensation section, which indicates the necessity to ensure the stability of the algorithm using small-signal stability theory [22,31].

The finite element method (FEM) is a numerical algorithm for the computation of the physical field, recognizing the most important tool for precise electromagnetic analysis. FEM helps to establish an accurate electromagnetic model of magnetic bearings, representing the relationship between inductance and the air gap, as well as the impact of non-ideal factors on displacement estimation. In [42,58], FEM was used to optimize the coupled magnetic network model of an 8-pole radial magnetic bearing. In [43], a three-pole radial magnetic bearing was analyzed using FEM to investigate the model of the current-induced magnetic saturation and the impact of the saturation on inductance estimation. Additionally, inductances were measured in experiments using an H-bridge excitation circuit, providing a comparison between experimental data and FEM simulation results.

Another approach to accounting for magnetic saturation is based on iterative magnetic circuit analysis utilizing the B-H curve. Since magnetic flux density and magnetic permeability are interdependent, the equivalent magnetic circuit is also considered nonlinear, which can be resolved by iteratively estimating the real-time saturation level, yielding the specific reluctance of the ferromagnetic core. The magnetic circuit iterative calculation method is more accurate than the polynomial model presented in Equation (3), but its complexity also increases significantly, primarily in the following aspects:

1. Modeling complexity: The geometric parameters and leakage magnetic coefficients of each segment of the magnetic circuit in the magnetic network are unclear, requiring interactive verification using FEM and various measured data, or even the construction of a nonlinear optimization solver. The design process is cumbersome, and model errors are difficult to locate;
2. Complexity of real-time computation: Magnetic circuit models exhibit high dimensionality and multi-parameter characteristics, making the iterative solution of the equation system unacceptable in real-time control scenarios.

In addition, there is an avoidance strategy involving prioritizing information from the actuators with lighter saturation or no saturation to mitigate its effect. For a 1-DOF magnetic bearing, an appropriate bias current can be selected to ensure that at least one side of the differential AMB maintains a lower magnetic flux density, thereby preventing it from entering the saturation region [44]. The information from this side is subsequently utilized as the primary reference or assigned a greater weight. For an eight-pole radial magnetic bearing, dynamic weighting factors can be introduced to adjust the contribution of each pole to the displacement estimation [42]. This approach ensures that the actuator with the lowest magnetic flux density dominates the estimation, thereby suppressing the adverse effects of saturation.

**Figure 6:**
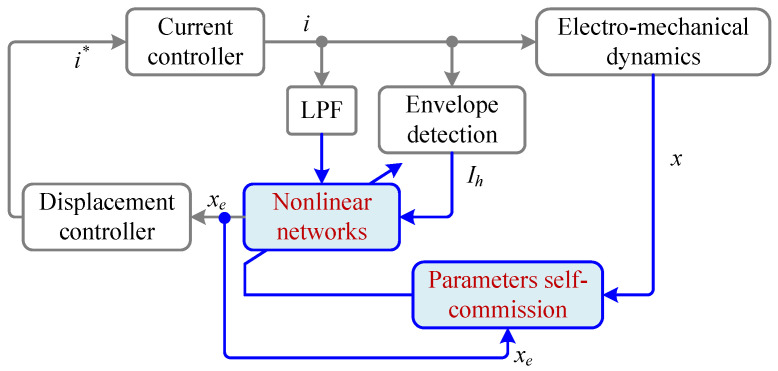
Block diagram of data-driven self-tuning self-sensing system.

The above-mentioned approaches are model driven, and establish magnetic saturation models by combining physical modeling with measured data to optimize parameters. From another perspective, data-driven approaches do not predefine analytical models for magnetic saturation. Instead, they employ network structures with both fitting and generalization capabilities to represent the relationship between measured variables and air gaps. By inputting measured data and applying certain learning algorithms, the model automatically adjusts its parameters to obtain an input–output mapping. The block diagram of the data-driven nonlinear compensation self-sensing control system is shown in Figure 6. The nonlinear networks are used to fit the mapping between measured variables and displacement, while its parameters are updated based on a learning algorithm that serves the network output error as the input. This step is completed in an offline process. When running online, the well-trained network is directly used as a displacement estimator. Data-driven methods can rely entirely on measured data for modeling, avoiding errors caused by oversimplification in theoretical analysis. However, data-driven methods require a large amount of test data, have low physical interpretability, involve significant debugging effort, and lack theoretical guarantees for stability and reliability.

### 3.2. Modeling and Online Correction of Eddy Current

The eddy current effect is caused by a high-frequency alternating magnetic field that induces currents within the conductor core, opposing changes in magnetic flux. The consequences of eddy currents include a reduction in inductance and equivalent permeability. Since the eddy currents introduce additional reluctance, they decrease the sensitivity of inductance to air gap variations. Additionally, eddy currents can cause nonlinear distortion in the PWM current ripple waveform, which serves as the self-sensing signal, primarily manifesting as alterations in waveform shape and phase lag. Therefore, they adversely affect the feasibility of displacement self-sensing.

To derive the model of electromagnets with eddy current effects, Meeker et al. use the Stoll one-dimensional eddy current model to analyze the eddy current effects of laminated cores [46], and derive the frequency-dependent complex permeability $\mu_{r}\left(s\right)$, whose expression is given as

(4) $$\mu_{fd}\left(s\right)=\mu \left[\frac{\tan h\left(\sqrt{s\sigma \mu }\frac{d}{2}\right)}{\sqrt{s\sigma \mu }\frac{d}{2}}\right]$$

where *d* is the lamination thickness, σ the electrical conductivity, μ the material permeability, and *s* the complex frequency that equals jω. Further, by expanding the hyperbolic tangent function using a continued fraction, the ferromagnetic reluctance is decomposed into an equivalent multi-stage resistor–inductor network. In [47], a three-level linear lumped RL network is used to fit the eddy current model from 0 to 20 kHz. This model accurately reproduces the decrease in inductance amplitude and phase recovery phenomenon in the high-frequency range while retaining the algebraic equation form of circuit theory, resulting in lower computational costs. In [59], an eddy current modeling method based on virtual lumped coils is proposed, using a fictitious single-turn coil model to simulate the eddy current path. The skin depth parameter δ was calibrated using experimental data, ensuring the model accurately repeats the waveform distortion caused by actual eddy currents. Based on this, a high-fidelity switch PWM-driven self-sensing AMB simulation model is proposed, providing a parameterized simulation platform for studying the effects of eddy currents on displacement self-sensing under different operating conditions.

The concept of complex permeability proposed by Meeker is adopted in [42]. However, to reduce the computational burden, an experimentally calibrated lookup table is embedded instead of the equivalent RL network model. By collecting voltage and current waveforms of the magnetic bearing under different average currents, the frequency characteristics of impedance were extracted using FFT, and the complex permeability was converted into a lookup table based on the estimated magnetic field strength B and embedded into the magnetic resistance network model. In [48], the amplitude-phase errors caused by the fractional-order dynamics of eddy currents are analyzed. Then, two methods to reduce the influence of eddy currents in self-sensing AMBs are proposed as follows:

1. Optimizing the magnetic circuit design, using large air gaps and the large pole area to optimize the electromagnetic coupling coefficient.
2. Real-time compensation method for the controller: constructing a fractional-order dynamic compensator, and identifying the eddy current coefficient and embedding it into the real-time controller.

In [36,49], a compensation method for laminated cores combining fundamental PWM ripple demodulation is proposed. By constructing a single-turn virtual winding model to analyze the PWM ripple current offset, real-time correction is introduced into the fundamental amplitude demodulation. In [60], magnetic resistance is divided into static magnetic resistance and dynamic magnetic resistance, and frequency-dependent dynamic magnetic resistance can be used to compensate for the effects of eddy currents.

## 4. Inductance Estimation Based on Amplitude Demodulation

### 4.1. Structures of Demodulator

Sensing signals can originate from the actuator itself—PWM ripple of the switching power amplifier or from an active injected high-frequency voltage signal. The PWM ripple scheme faces dynamic compensation issues, meaning that when the control component changes, the amplitude of the PWM ripple also changes. Changes in ripple amplitude result in sideband components at the sensing frequency, necessitating appropriate duty cycle compensation strategies [23,36]. Actively injecting high-frequency signals can avoid the issue of varying sensing component amplitude during system dynamics, eliminating the need for duty cycle compensation.

**Figure 7:**
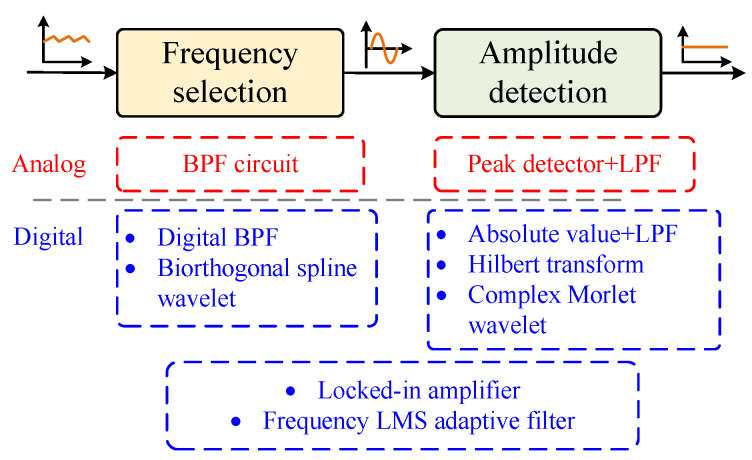
General block diagram of amplitude demodulation methods.

Amplitude demodulators are generally divided into two parts: the first part extracts the single-frequency sensing component from the current and voltage, functioning as a frequency-selective network, while the second part extracts the amplitude of the sensing component. For both of these stages, there are various analog or digital implementation schemes available. The general block diagram of the amplitude demodulator can be represented by Figure 7.

The frequency selection stage can employ an analog bandpass filter, which combines a filtering function with signal amplification. In PWM ripple demodulation, considering the spectral changes caused by dynamic processes, the amplitude demodulator should extract the components of the PWM frequency and its primary sideband frequencies. This requires the BPF to have a wide and flat passband, making the Butterworth filter more suitable for amplitude demodulation. If a digital form of the filter is used, lower-order infinite impulse response (IIR) filters are preferred for real-time control to reduce computational burden. It is worth noting that detecting high-frequency micro signals poses a challenge for limited ADC resolution. Therefore, an appropriate analog front-end amplifier should be used to amplify the high-frequency components before detecting them.

The amplitude detection can be implemented using a precise rectifier circuit. To obtain a smooth signal amplitude, it must also pass through a low-pass filter. This method directly maps to the digital domain as an absolute value function and digital low-pass filter (LPF). The output of this configuration is the periodic average value of the rectified signal, which is 2/π of the actual amplitude.

Analog demodulators achieve demodulation through an analog circuit, which helps alleviate computational load, thereby freeing up processor resources for executing magnetic levitation control algorithms. Despite this advantage, analog demodulators suffer from several notable drawbacks compared to their digital counterparts. Firstly, they substantially increase hardware cost and complexity while reducing overall system reliability. Moreover, their performance is constrained by inherent non-idealities, such as component parameter drift and the forward voltage drop in diodes, which ultimately limit estimation accuracy. In contrast, digital demodulators are implemented in software, offering fully configurable parameters and structures. This programmability significantly enhances the flexibility of the displacement estimation stage, making digital approaches the prevailing trend.

Other digital implementations like Hilbert transformation and complex Morlet wavelet transformation are also mentioned by articles [33]. The essence of the Hilbert transform is an all-pass filter, which constructs the imaginary part of the signal through an orthogonal transform to achieve a complete representation in the complex domain. An *N*th-order finite impulse response (FIR) filter can be used to approximate the ideal Hilbert transformation. Additionally, the input signal must be delayed by N/2 cycles to compensate the fixed group delay introduced by the linear phase.

The complex Morlet wavelet defines filtering behavior from a convolution perspective and can be analogized as a series of BPFs. However, the convolution kernel varies with the translation parameter and is essentially a time-varying system. Therefore, it cannot be expressed as a transfer function like an LTI filter. The analysis of its filtering characteristics should be based on time-domain waveform similarity rather than a fixed frequency-domain response.

**Figure 8:**
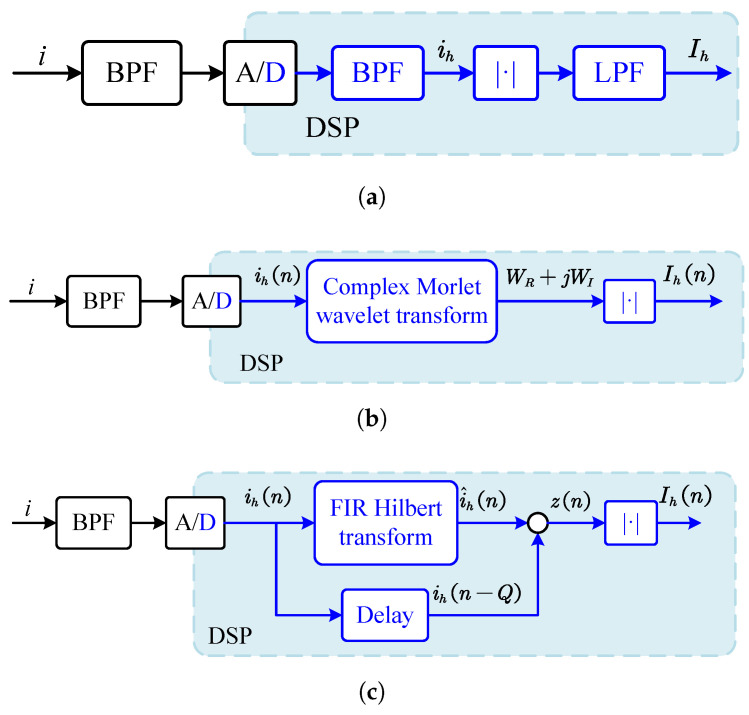
Block diagram of three demodulation schemes. (**a**) Absolute value. (**b**) Complex Morlet wavelet. (**c**) Hilbert transform.

Figure 8 shows the block diagram of several amplitude demodulators based on digital algorithms. Hilbert transform and wavelet transform adopt high-order FIR filters or convolution operations, which are difficult to implement on a DSP platform and require a higher-performance hardware platform such as the FPGA.

### 4.2. Parametric Amplitude-Phase Extraction Algorithms

Besides the two-step implementation mentioned above, there are also algorithms that can precisely analyze and decompose the signal into various frequency components. Then, the amplitude and phase of the desired frequency component, such as phase-locked loops and frequency-domain LMS filters, can be directly calculated. These algorithms do not require the configuration of BPF, as the algorithms themselves have the filtering capability. Additionally, the amplitude is contained in the parameters, eliminating the necessity for the LPF at the output. Therefore, theoretically, this reduces digital complexity and minimizes the delay introduced by filters.

**Figure 9:**
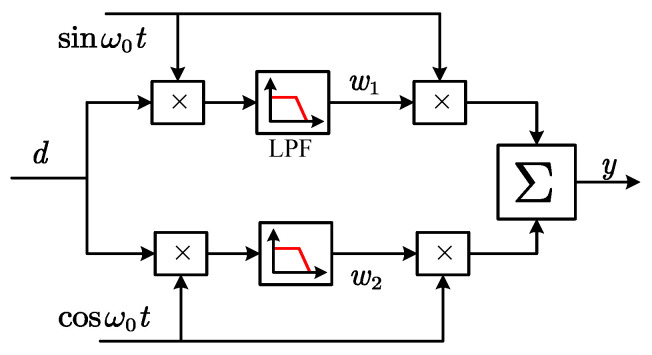
Schematic diagram of the locked-in amplifier.

The locked-in amplifier (LIA) operates based on the principle of orthogonality between trigonometric functions at different frequencies. The input signal is multiplied by a reference signal at the target frequency, whereby only the component at this specific frequency contributes to the DC portion of the resulting product. Therefore, by applying a low-pass filter subsequently, the amplitude of the target frequency component can be extracted. Since the multiplication (mixing) of signals generates both sum and difference frequency components, the suppression capability of the LPF at twice the sensing frequency requires particular attention. The schematic block diagram of the lock-in amplifier is shown in Figure 9. Using the Fourier transform, the frequency response characteristics of the LIA can be derived as

(5) $$G_{\mathrm{LIA}}\left(s\right)=\frac{1}{2}\left[g_{f}\left(s+j\omega_{0}\right)+g_{f}\left(s-j\omega_{0}\right)\right]$$

where $g_{f}\left(s\right)$ is the transfer function of the LPF and $\omega_{0}$ is the sensing frequency. As the cutoff frequency of the LPF decreases, the filtering performance improves, but the phase shift increases. Meanwhile, the group delay at the center frequency of the passband also increases, indicating a decline in dynamic performance. The amplitude of the sensing signal is $\sqrt{w_{1}^{2}+w_{2}^{2}}$, while this result is not equal but half of the amplitude.

**Figure 10:**
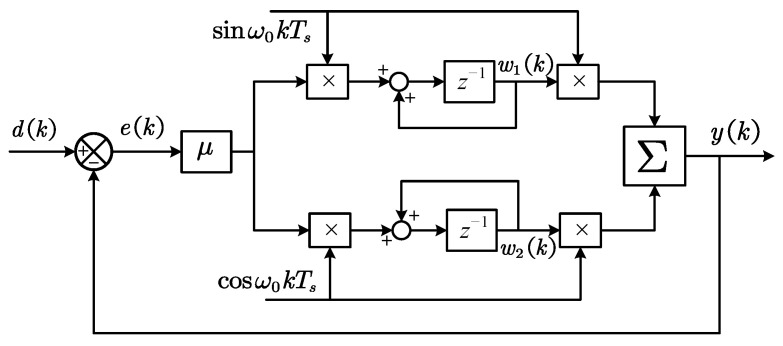
Schematic diagram of the LMS filter.

The frequency-domain LMS filter, serving as an enhanced variant of adaptive filters, has found extensive application in real-time signal processing since its introduction by Widrow in 1976 [61]. The LMS filter updates its parameters based on the minimum mean square error principle, thereby reconstructing the desired frequency signal. The schematic diagram of the LMS adaptive filter is illustrated in Figure 10, and its state-space representation can be formulated as

(6) $$\begin{array}{c} y\left(k\right)=w_{1}\left(k\right)\cos\left(\Omega kT_{s}\right)+w_{2}\left(k\right)\sin\left(\Omega kT_{s}\right)\\ w_{1}\left(k+1\right)=w_{1}\left(k\right)+\mu e\left(k\right)\cos\left(\Omega kT_{s}\right)\\ w_{2}\left(k+1\right)=w_{2}\left(k\right)+\mu e\left(k\right)\sin\left(\Omega kT_{s}\right) \end{array}$$

The discrete impulse transfer function can be derived as

(7) $$G\left(z\right)=\frac{\mu \left(z\cos\Omega T_{s}-1\right)}{z^{2}+z\cos\left(\Omega T_{s}\right)\left(\mu -2\right)+1-\mu }$$

**Figure 11:**
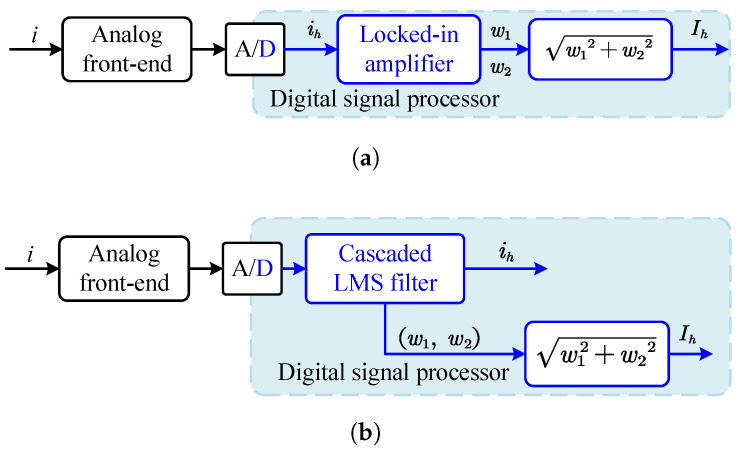
Block diagram of the demodulators based on two algorithms. (**a**) Locked-in amplifier. (**b**) LMS filter.

Among these, the parameter μ influences the frequency characteristics and the dynamic performance. The smaller the μ, the narrower the passband and the stronger the frequency selection capability, but the dynamic response decreases. Therefore, it is necessary to balance the performance of both aspects and select the parameters reasonably. The block diagrams of demodulators based on the LIA and the LMS filter are shown in Figure 11. Table 2 presents the key parameters of various digital demodulation algorithms and their impact on demodulator performance.

### 4.3. Performance Evaluation and Comparison

Some performance metrics of self-sensing AMBs utilizing different demodulation approaches described in the presented articles can be seen in Table 3. Then, simulations were conducted to compare the performance of the displacement self-sensing systems. For all the demodulators discussed, the high-frequency signal is uniformly injected to avoid the influence of the dynamic duty cycle. The parameters of the system are shown in Table 4. Several representative demodulator types were selected for evaluation:

(1) Analog BPF + absolute value + digital LPF.
(2) Digital locked-in amplifier.
(3) Frequency LMS filter.

**Figure 12:**
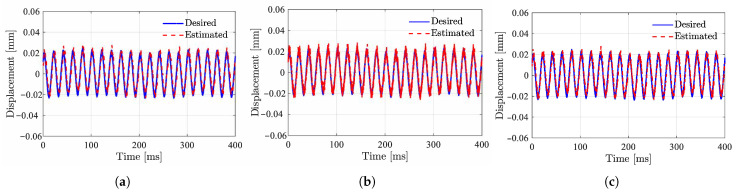
Measured and estimated displacements at the rotor speed of 50Hz. (**a**) Configuration (1). (**b**) Configuration (2). (**c**) Configuration (3).

In configuration (1), a Butterworth BPF is used. In configurations (2) and (3), to avoid the sampling distortion of small high-frequency signals due to insufficient AD resolution, a high-pass filter is added to amplify high-frequency signals and suppress low-frequency control signals. To conduct an optimal dynamic performance, for designing each demodulator, this paper chooses the structure that confirms the fastest dynamic response to the displacement variation, under the constraint that the noise signal in the estimated displacement is confined to a specific threshold. Figure 12 shows the waveforms of the estimated displacement and actual displacement by simulations. The system is subjected to an external disturbance of 50 Hz, corresponding to the excitation by mass unbalance at a speed of 3000 rpm. From this, the accuracy of the displacement estimation values is satisfactory with small errors and high signal-to-noise ratios.

**Figure 13:**
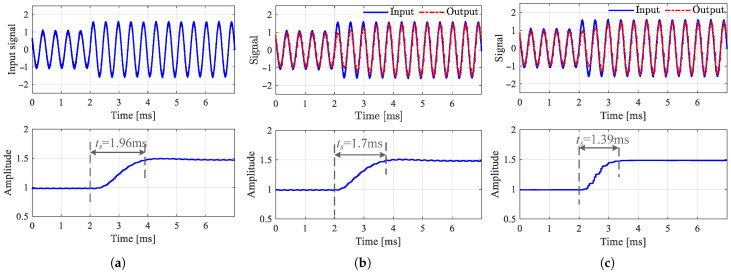
Step response of selected demodulator. (**a**) Configuration (1). (**b**) Configuration (2). (**c**) Configuration (3).

**Figure 14:**
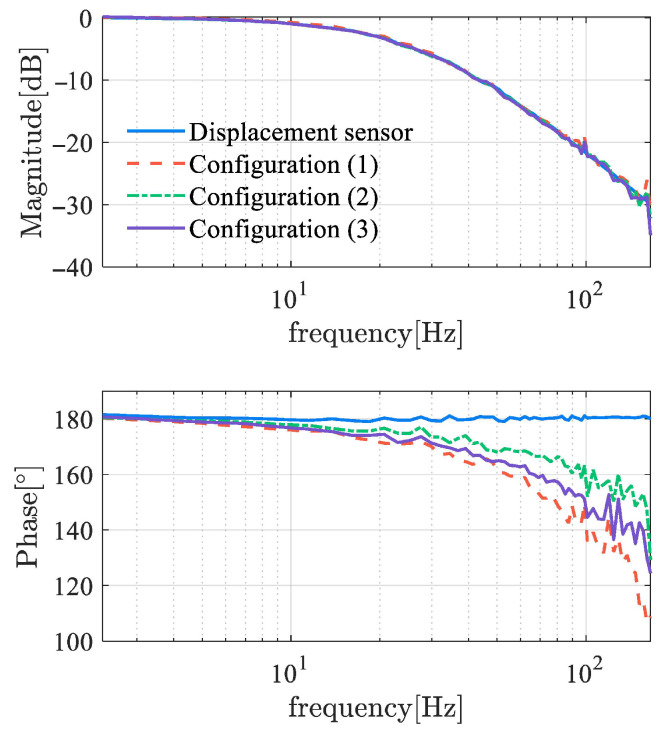
Frequency response function of self-sensing AMB based on demodulation.

The evaluation of the dynamic response time of the demodulator is conducted from multiple aspects. As shown in Figure 13, it is an offline test of the demodulators that examines the response time of the demodulators to track the step amplitude. The step setting times of several demodulators are all in the ms range, indicating a fast response speed. Using frequency domain identification theory, Figure 14 shows the frequency response function (FRF) of self-sensing AMBs, where the input of the tested object is the control current $i_{c}$, and the output is the estimated displacement. An AMB with displacement sensors has a constant 180° phase, while the phases of self-sensing systems have a certain delay. The phase delay of the self-sensed AMB plant increases with frequency, while the amplitude of that is basically consistent with the original sensor-based system. This indicates that since the cutoff frequency of the self-sensing system remains the same as the sensor-equipped system, phase lag can lead to a reduction in phase margin and may even cause instability. Obviously, the phase margin loss achieved using the demodulation-based method is significantly improved compared to the observer method, resulting in enhanced stability.

**Figure 15:**
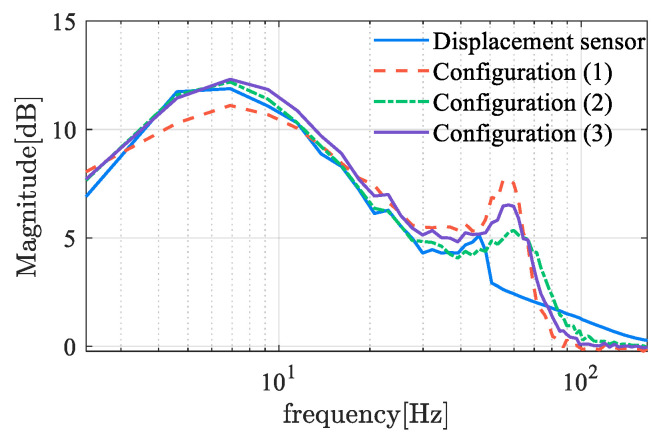
Sensitivity function of self-sensing AMB based on demodulation.

Examine the sensitivity function, which is defined as

(8) $$S\left(s\right)=\frac{1}{1+L\left(s\right)}$$

where L(s) represents the open-loop transfer function. The sensitivity function reflects the system’s sensitivity to disturbances and model perturbations. A smaller amplitude-frequency characteristic indicates stronger robustness. Figure 15 shows the frequency characteristics of the sensitivity functions for self-sensing AMBs. The amplitude reaches the sensitivity peak at approximately 7 Hz, with a peak value of about 11–13, which indicates the robustness of B and C classes. However, magnetic bearings typically operate at a high frequency, where the amplitude of sensitivity is relatively small. There is an evident difference between self-sensing systems and sensor-equipped systems at the range of 50 to 80 Hz, where the sensor-equipped system exhibits better robustness. This distinction between 50 Hz to 80 Hz is primarily attributable to the time delay inherent in the displacement estimation process. As the delayed time increases, the second amplitude peak is observed to shift toward a lower frequency domain, accompanied by an increase in peak value. This demonstrates the significant deterioration in performance that occurred when utilizing displacement self-sensing in comparison with the displacement sensor.

In Table 5, the phase margin under the PID controller and the $H_{\infty }$ norm of the sensitivity function at typical operating frequencies are listed. The robustness of displacement self-sensing systems dominated by three demodulation algorithms is similar in terms of sensitivity metrics. However, the stability and time delay show some differences, with performance ranked as follows: locked-in amplifier, LMS filter, and absolute value detection algorithm.

To provide a comprehensive comparison, the relative merits and limitations of various digital demodulation algorithms are discussed herein, with the critical premise that all are designed for equivalent noise suppression and filtering performance. The BPF combined with absolute value detection and LPF is characterized by its implementation simplicity; however, this comes at the expense of significant phase delay and a consequently reduced stability margin. In contrast, the Hilbert transform achieves superior demodulation accuracy, yet it inherently introduces a large group delay due to its high filter order, a limitation that can only be mitigated by implementing a high sampling rate. Similarly, the Morlet wavelet transform offers exceptional time–frequency localization and thus very high precision, but its demanding computational load from continuous convolution also necessitates both high processing power and a high sampling rate for dynamic applications. Meanwhile, the locked-in amplifier stands out for its excellent accuracy, minimal data storage, straightforward implementation, and fast dynamic response, although its performance is contingent upon the effective design of the output low-pass filter to suppress components at twice the carrier frequency. Conversely, the LMS filter features rapid dynamic response, high accuracy, and a simple structure, but a single LMS filter struggles to simultaneously achieve satisfactory noise rejection and dynamic performance, often requiring a more complex cascaded design.

Consequently, for general-purpose control platforms, the locked-in amplifier and LMS filter present a favorable balance, enhancing dynamic performance without sacrificing noise immunity. On advanced hardware platforms that support high sampling rates and substantial computational throughput, the Hilbert and Morlet wavelet transforms emerge as viable alternatives where utmost precision is required.

## 5. Inductance Estimation Based on Least Squares Identification

Parameter identification relies on continuous-time sampling to construct the identification equation. For the system in a steady state, there exists a rank deficiency in the identification equation. The rank deficiency means that there are multiple linearly correlated rows in the regression matrix, resulting in the rank of the regression matrix being less than the number of parameters to be identified, and there are infinite solutions. Typically, the dynamics of the excitation system are required to ensure the identifiability of the parameters. The excitation signal can still be generated using PWM switching harmonics and additional high-frequency voltage injection.

### 5.1. Construction and Solution of Identification Equations

**Figure 16:**
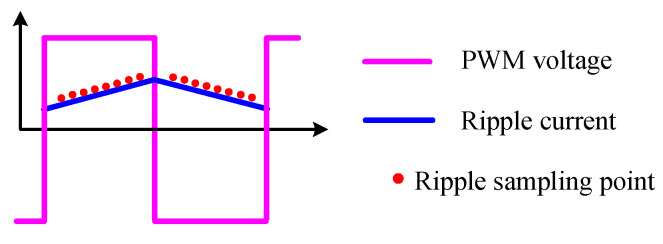
Waveforms of PWM voltage and ripple current.

The voltage of a PWM amplifier is in the form of pulses with variable width. If bipolar modulation is used, the output voltage and current waveforms are as shown in Figure 16. It can be seen that within a PWM cycle, the current exhibits exponential rise and fall phases. Considering some reasonable simplification, the current waveform can be approximated as linear. During the rise and fall phases of the current within a PWM cycle, ripple current is sampled at specific time points, with the sampling time and value recorded as

(9) $$\left\{\begin{array}{c} t_{r}=\left[t_{r1},\;\;t_{r2},\;\;\cdots ,\;\;t_{r.m}\right]\\ t_{f}=\left[t_{f1},\;\;t_{f2},\;\;\cdots ,\;\;t_{f.n}\right]\\ i_{r}=\left[i_{r1},\;\;i_{r2},\;\;\cdots ,\;\;i_{r,m}\right]\\ i_{f}=\left[i_{f1},\;\;i_{f2},\;\;\cdots ,\;\;i_{f,n}\right] \end{array}\right.$$

where the subscript *r* represents the rising phase, *f* is the falling phase, and *m* and *n* are sampling numbers during the rising and falling phases, respectively. The sampling time should avoid overlapping with the switching instances and maintain a certain interval from them. The reason is that the EMI is strongest at the switch instances, which can easily affect the reliability of current sampling. Moreover, eddy currents can cause severe distortion of the current waveform near the switching instances.

The identification function can be derived from the discrete form of the flux equation, which is given as

(10) $$\underset{y}{\underset{︸}{\left[\begin{array}{c} i_{r2}\\ i_{r3}\\ \vdots \\ i_{rm} \end{array}\right]}}=\underset{\Phi }{\underset{︸}{\left[\begin{array}{cc} 1 & \Delta \psi_{r1}\\ 1 & \Delta \psi_{r2}\\ \vdots & \vdots \\ 1 & \Delta \psi_{r,m-1} \end{array}\right]}}\underset{\theta }{\underset{︸}{\left[\begin{array}{c} i_{r1}\\ L^{-1} \end{array}\right]}}$$

where θ contains the parameters to be estimated, and Φ and y are the regression matrix and measurement vector. Δψ is the change in flux, given as

(11) $$\Delta \psi_{rk}=T_{s}\sum_{j=1}^{k}\left(V_{dc}-Ri_{rj}\right)$$

and the solution in the least squares sense is given by the pseudo-inverse as

(12) $$\hat{\theta }={\left(\Phi^{T}\Phi \right)}^{-1}\Phi^{T}y$$

Since the dimension of the equation is relatively low, the above equation can lead to the solution given as

$${\hat{L}}_{r}=\frac{\left(m-1\right)\sum_{k=1}^{m-1}{\left(\Delta \psi_{rk}\right)}^{2}-{\left(\sum_{k=1}^{m-1}\Delta \psi_{rk}\right)}^{2}}{\left(m-1\right)\sum_{k=1}^{m}\left(i_{rk+1}\Delta \psi_{rk}\right)-\left(\sum_{k=1}^{m-1}\Delta \psi_{rk}\right)\left(\sum_{k=1}^{m}i_{rk+1}\right)}$$

(13) $${\hat{L}}_{f}=\frac{\left(n-1\right)\sum_{k=1}^{n-1}{\left(\Delta \psi_{fk}\right)}^{2}-{\left(\sum_{k=1}^{n-1}\Delta \psi_{fk}\right)}^{2}}{\left(n-1\right)\sum_{k=1}^{n}\left(i_{fk+1}\Delta \psi_{fk}\right)-\left(\sum_{k=1}^{n-1}\Delta \psi_{fk}\right)\left(\sum_{k=1}^{n}i_{fk+1}\right)}$$

which is the direct estimation equation. This is efficient in reducing the numerical errors introduced by solving the linear equation. There is also a method that takes into account two types of errors: resistance mismatch and changes in inductance values within a PWM cycle. By combining the currents from the rising and falling segments, improved estimation has been proposed [39].

**Figure 17:**
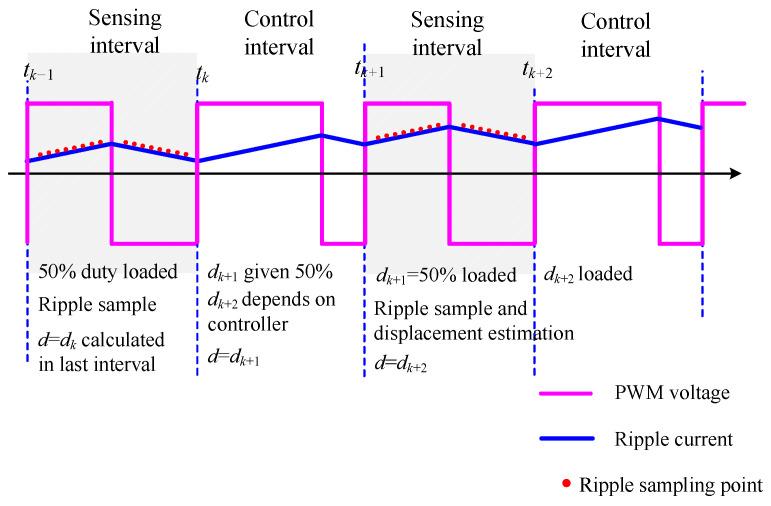
Digital implementation showing measurement and control cycles.

The design of a digital program for PWM ripple detection seems quite challenging. This challenge manifests in two aspects. First, the PWM switching frequency and the current sampling frequency are inconsistent. Second, as the duty cycle changes, the current sampling instances also need to be adjusted. One solution to deal with the ripple sampling under dynamic duty cycles is to set alternating control and measurement cycles [31], with the digital implementation as shown in Figure 17.

If high-frequency voltage injection is adopted, the control voltage is represented as $u^{\prime }=u_{c}+U_{h}\sin\left(\omega_{h}t\right)$, where $u_{c}$ is the current controller outputs, and $U_{h}$ and $\omega_{h}$ are the amplitude and angular frequency of the injected signal. Compared with the ripple detection method, the sensing signal frequency of the high-frequency injection method is constant and not affected by dynamic duty cycles. Therefore, the sensing component can be separated and amplified first, and then sampled, reducing the impact of ADC resolution and sampling noise. In addition, the switching frequency and sampling frequency are equal, which allows the current sampling to be triggered by hardware, making software implementation more convenient.

### 5.2. Performance Evaluation and Comparison

The PWM frequency and injection frequency of the high-frequency injection method are the same as those in the previous section. Since the sensing signal has a fixed frequency, it is amplified by the analog front-end. The PWM ripple detection method uses a switching frequency of 5 kHz and the bipolar modulation strategy, and the DC-link voltage is increased to 100 V.

In the ripple detection method, there are parameters that significantly affect the precision of estimation results. These include the DC voltage $V_{dc}$, the PWM frequency $f_{pwm}$, and the number of samples during the current rise and fall phases. The sampling noise is set as a Gaussian white noise sequence with a mean of 0. The standard deviation σ is given in the form of the integer value of 12-bit AD conversion noise. The noise of the parameter identification results is defined as

(14) $$E_{L}=\exp\left(\frac{\left|\hat{L}-\bar{L}\right|}{\bar{L}}\right)\times 100\%$$

**Figure 18:**
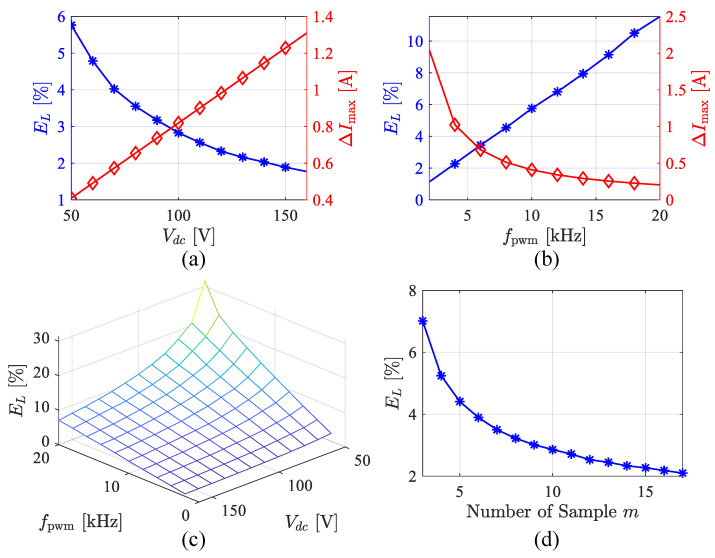
Average noise versus multiple parameters. (**a**) DC-link voltage $V_{dc}$. (**b**) PWM frequency $f_{pwm}$. (**c**) $V_{dc}$ and $f_{pwm}$. (**d**) Number of samples *m*.

The above equation represents the mathematical expectation of the relative error, with the mean value replacing it in a statistical sense. Figure 18 illustrates the influence of these parameters on the inductance estimation error. Figure 18a shows the variation in ripple amplitude and noise with respect to the DC voltage. Obviously, as the DC voltage increases, the mean error decreases. The relationship between noise and PWM frequency is shown in (b). Noise generally increases linearly with PWM frequency. Considering the combined effects of Vdc and fpwm on EL, (c) shows the variation in the form of a surface. (d) illustrates the impact of the number of samples on EL. It is evident that as m and n increase, EL decreases, indicating that the SNR improves. These results provide guidance for parameter selection.

**Figure 19:**
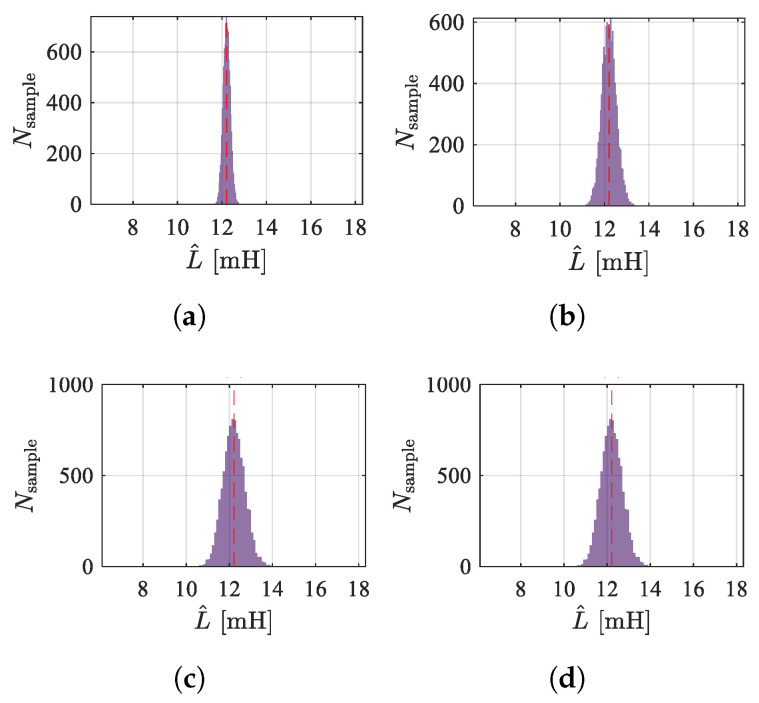
Distribution of estimation results by PWM ripple method under different sampling noise conditions. (**a**) σ=5. (**b**) σ=10. (**c**) σ=15. (**d**) σ=20.

**Figure 20:**
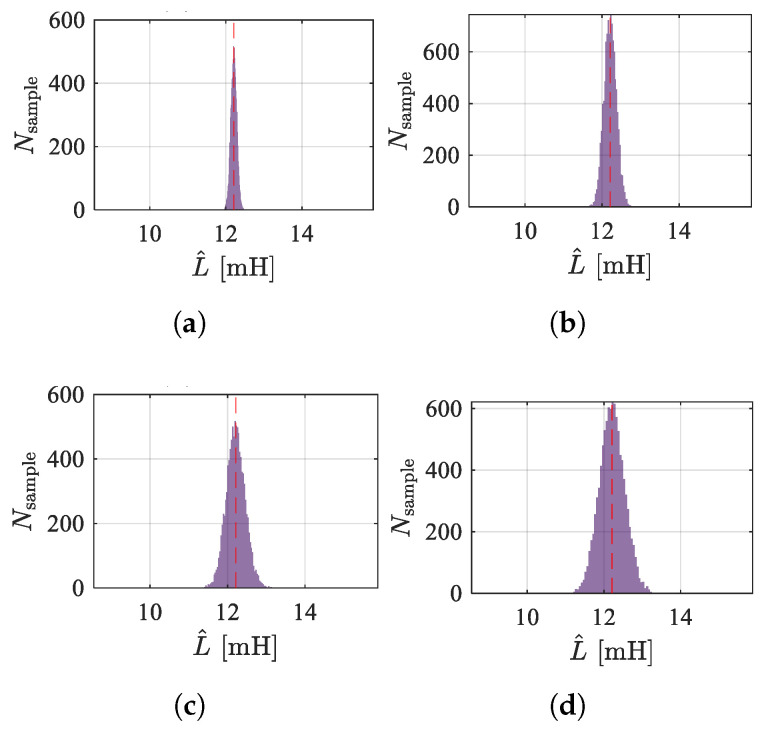
Distribution of estimation results by injection method under different sampling noise conditions. (**a**) σ=5. (**b**) σ=10. (**c**) σ=15. (**d**) σ=20.

**Figure 21:**
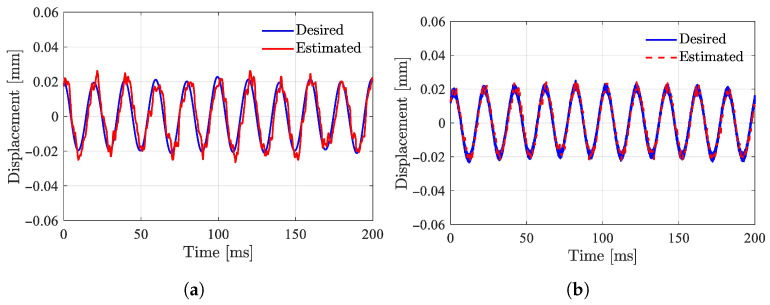
Displacement estimated using least squares identification. (**a**) PWM ripple based. (**b**) HF injection based.

Set the standard deviation of sampling noise from 5 to 20. Figure 19 and Figure 20 show the frequency distribution histograms of the inductance estimates for the two methods. As the noise increases, the distribution of inductance estimates becomes more dispersed, and the SNR decreases. Due to the amplification of high-frequency signals, the high-frequency injection method is less affected by sampling noise. However, in terms of the mean value, the PWM ripple method is closer to unbiased estimation, which is related to the sampling rate and the accuracy of the discretization model. Figure 21 shows the estimated displacement waveforms under a 50 Hz disturbance (3000 rpm speed).

**Figure 22:**
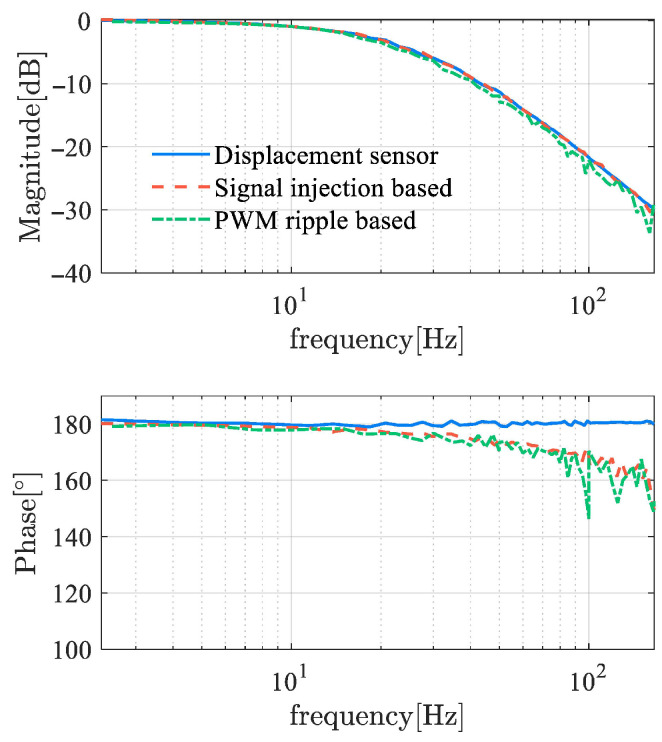
Sensitivity function of self-sensing AMB based on demodulation.

**Figure 23:**
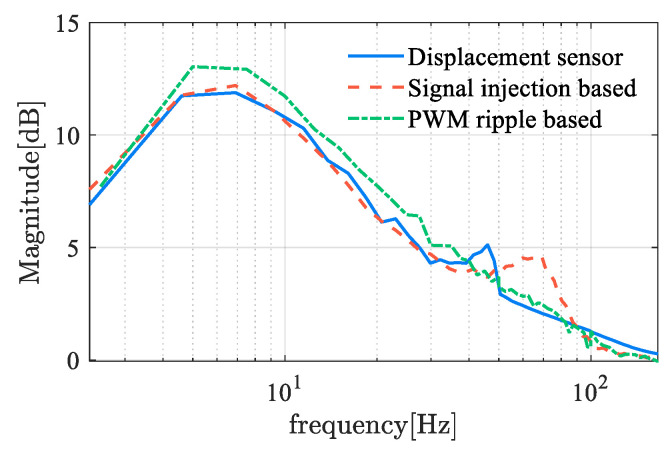
Frequency response function of self-sensing AMB based on demodulation.

Robustness and stability are evaluated based on the FRF and sensitivity function of the controlled system. The FRF of the controlled plant is illustrated in Figure 22. The phase lag of the PWM ripple-based method is more pronounced, which can be attributed to the following rationale. Theoretically, the ripple detection approach completes the estimation within two PWM cycles, thereby achieving a very high bandwidth. However, the introduction of a low-pass filter to enhance the signal-to-noise ratio introduces additional phase delay. This reflects a fundamental trade-off between signal-to-noise ratio and dynamic response speed, necessitating a balanced design in practice. Typically, the parameter estimation value is processed through an LPF to achieve a smoother waveform. The LPF plays an essential role in the trade-off between SNR and dynamic performance. The cutoff frequency of the LPF is generally selected as the maximum value that still satisfies the SNR requirement, as this configuration ensures the fastest possible dynamic response under the given SNR constraint. The phase stability margin of the displacement self-sensing system is computed and summarized in the accompanying table. Furthermore, the robustness metric, defined in terms of the sensitivity function, is provided in Figure 23 and Table 6.

In summary, the high-frequency injection method demonstrates lower sensitivity to sampling noise than the PWM ripple method. When the noise level in the estimation output is constrained to a comparable range through a post-processing LPF, the high-frequency injection approach still achieves a larger phase margin and superior dynamic performance. In contrast, the PWM ripple method is subject to several practical limitations. For example, excessively high electromagnet inductance can attenuate the current ripple amplitude to an unmeasurable level. Additionally, in the absence of laminated cores, eddy currents can severely distort the PWM current waveform, making the ripple-based estimation impractical. Therefore, this study concludes that the high-frequency injection method exhibits better overall performance and wider applicability than the PWM ripple method.

Based on simulation analysis between the demodulation method and the parameter identification method, the following conclusions can be drawn. When the noise levels in the estimation results are comparable, the least squares parameter identification approach exhibits superior dynamic performance over the demodulation method. This advantage is reflected in its smaller phase lag, larger stability margin, and lower upper bound of the sensitivity function. It should be noted, however, that the parameter identification method shows higher sensitivity to disturbances such as resistance mismatch, PWM dead-time effects, and current sampling noise. In practice, demodulation remains a more mature and widely adopted solution. Nevertheless, for applications requiring high dynamic performance, parameter identification represents a promising direction for future development.

## 6. Tendency Discussion

### 6.1. Improvements in Dynamic Performance

Existing research indicates that the phase lag of self-sensing AMB systems increases significantly with frequency, which severely limits the performance of high-speed rotor systems. To overcome this, solutions can be sought from two dimensions: hardware optimization and algorithm improvement. On the hardware aspect, system bandwidth can be expanded by optimizing the demodulator design or adopting higher-frequency sensing signals. However, analog circuits are constrained by the structure of the filter and rectifier circuit, facing inherent limitations. Future trends may involve the deep integration of high-speed data detection technology (such as high-precision ADCs) with high-performance computing platforms (DSP+FPGA architectures). By the extraction of the sensing signal and combining advanced digital demodulation algorithms like adaptive filter and wavelet analysis, the dynamic response of the system can be enhanced.

### 6.2. Robustness and Disturbance Attenuation

The self-sensing technology faces several challenges in enhancing robustness and attenuating disturbance. Research indicates that the eddy current causes distortion of the triangular current ripple, thereby narrowing the effective detection window. Further research requires solutions from two dimensions: material optimization and algorithm compensation. From the first perspective, the use of low eddy current loss materials, such as optimally designed laminated core, can effectively suppress eddy current effects. At the algorithm level, advanced compensation algorithms can improve displacement estimation quality.

Additionally, refining the inductance model is another key point to improve displacement sensing accuracy, requiring comprehensive consideration of nonlinear factors such as saturation and temperature drift. Development trends will focus on developing adaptive algorithms that adjust inductance model parameters in real time to achieve continuous optimization of accuracy. This technical approach, which integrates both material and algorithmic innovation, holds promise for enhancing the robustness of displacement self-sensing systems under complex operating conditions.

### 6.3. Integration with Intelligent Algorithms

AMB displacement self-sensing technology is showing a trend toward the integration of intelligent algorithms. Cutting-edge research is actively exploring innovative applications of machine learning algorithms: by constructing nonlinear input–output models of actuators using machine learning methods (such as support vector machine and multi-layer neural networks), features can be directly extracted from raw current and voltage signals, effectively avoiding nonlinear errors introduced by simplifying assumptions in traditional analytical models. Another breakthrough is that data-driven algorithms can achieve adaptive fusion of parameters, updating key variables in real time through online learning mechanisms, thereby addressing model mismatch issues. This intelligent solution is providing a new technical paradigm for achieving high-precision, robust displacement self-sensing systems.

### 6.4. Innovation in Hardware

The hardware integration trend suggests that integrating sensing coils as redundant calibration units within self-sensing systems can enhance system reliability and robustness under extreme operating conditions.

Additionally, the high-frequency characteristics (up to MHz level) of wide bandgap semiconductor devices, including SiC and GaN, not only significantly reduce power consumption and temperature rise but also generate current ripple signals with superior spectral characteristics. Meanwhile, new power converter topologies, such as two-phase-three-arm configurations, can provide structural redundancy and fault-tolerant capabilities for self-sensing systems.

## 7. Conclusions

Driven by the demands for cost-effectiveness, high integration, and reliability in magnetic bearings, the development of high-precision displacement self-sensing AMBs with enhanced robustness has garnered increasing attention. This paper comprehensively examines the state of the art in displacement self-sensing technologies. A unified framework is first established to systematically categorize existing research efforts in this domain, elucidating the connections and distinctions among various approaches. Regarding the modeling, the linear magnetic circuit model is introduced, followed by nonlinear modeling strategies and online compensation techniques addressing critical nonlinearities including saturation and eddy current effects. The analysis procedure for displacement estimators based on demodulation and parameter identification methods is elaborated, with comparative simulations demonstrating their interrelationships.

Specifically, parameter identification schemes achieve a faster dynamic response, which contributes to improved closed-loop stability margins; however, they are more susceptible to noise in the sampled signals, necessitating a careful design trade-off between the signal-to-noise ratio and dynamic performance. In contrast, digital demodulation algorithms based on parametric amplitude-phase extraction can provide superior phase stability margins compared to traditional demodulators.

Building upon these findings, the paper discusses future trends, highlighting that intelligent algorithms are promising in addressing nonlinearities inherent in electromagnetic actuator models. Furthermore, hardware–software co-optimization—enabled by processors with higher sampling rates and enhanced computational capacity—will facilitate the adoption of more advanced frequency-domain analysis algorithms, thereby significantly improving the performance of displacement self-sensing systems. 

## Figures and Tables

**Table 1:** Sensitivity classification.

Zone	Sensitivity Peak [dB]	Description
A	peak < 8	commissioned AMB system
B	8 < peak < 12	acceptable for unrestricted long-term running
C	12 < peak < 14	unsatisfactory sensitivity, which is not suitable for long-term operation
D	Peak > 14	severe sensitivity that can lead to damage to the machine

**Table 2:** The parameters that contribute to demodulation performance and their impact on noise suppression.

Algorithm	Structures and Parameters	Effect of Parameter Increase on Noise Suppression
Absolute value demodulation	Order of the BPF	Improves
	Passband width of the BPF	Degrades
Hilbert transform	Order *N*	Improves
Morlet wavelet transform	Shape factor σ	Degrades
	Scale factor α	Unchanged
Lock-in amplifier (LIA)	Cutoff frequency of the internal LPF	Degrades
LMS filter	Damping coefficient μ	Degrades

**Table 3:** Quality metrics of different demodulation methods.

Articles	Demodulation Structure	Experimental Setup	Evaluation Results
9	Analog 2-order BPF Rectifier LPF	ADI AD835 divider (Wilmington, MA, USA) TI TL084 active filter (Dallas, TX, USA)	Self-sensing sensitivity is 2100 V/m Good linearity of position sensing Peak of sensitivity function is 14 dB at 85 Hz
34	Differential ripple amplifier Direct envelope measurement	High-speed ZOH ripple detector FPGA + DSP configuration	A maximum static estimation error of 20 μm Frequency response delay is larger than the conventional method Peak of sensitivity is 10 dB at 12 Hz
40	Digital IIR BPF Hilbert FIR filter	Intel EP4CE15 FPGA (Santa Clara, CA, USA) + TI 28335 DSP (Dallas, TX, USA)	Nonlinearity between estimated and actual displacement less than 5 Stable operation at the speed lower than 2000 rpm

**Table 4:** Parameters of the evaluated system.

Parameter	Value
Area of the poles Ap [mm^2^]	105
Number of poles	2
Turns of coil *N*	130
Nominal gap $g_{0}$ [mm]	0.4
Maximum gap $g_{m}$ [mm]	0.2
Inductance under nominal gap $L_{0}$ [mH]	12.2
Bias current $I_{0}$ [A]	1.0
DC-link voltage $V_{dc}$ [V]	46
PWM frequency $f_{s}$ [kHz]	23
Frequency of injection signal $f_{h}$ [kHz]	2.3
Proportional control parameter $K_{P}$	3250
Integral control parameter $K_{I}$	13,330
Differential control parameter $K_{D}$	17

**Table 5:** Stability and robustness metrics of demodulation-based displacement self-sensing AMB.

Performance	Position Measured	Configuration (1)	Configuration (2)	Configuration (3)
Phase margin [°]	40.9	25.3	34.2	29.7
Norm of sensitivity function at 3000 rpm [dB]	3.3	6.6	4.7	5.6
Norm of sensitivity function at 6000 rpm [dB]	1.3	-0.1	0.5	0.1

**Table 6:** Stability and robustness metrics of displacement self-sensing AMB based on least squares identification.

Performance	Position Measured	Demodulation	Signal Injection	PWM Ripple Based
Phase margin [°]	40.9	34.3	37.1	34.4
Norm of sensitivity function at 3000 rpm [dB]	3.3	4.7	3.9	3.5
Norm of sensitivity function at 6000 rpm [dB]	1.3	0.5	0.8	0.9
